# Intensive Induction in AML in the Era of Venetoclax: A Standing Count for “7 + 3”?

**DOI:** 10.1002/ajh.70389

**Published:** 2026-06-07

**Authors:** Sudhir Tauro, Ayalew Tefferi

**Affiliations:** ^1^ Division of Cancer Research University of Dundee Dundee UK; ^2^ Department of Haematology Ninewells Hospital & School of Medicine Dundee UK; ^3^ Division of Hematology, Department of Internal Medicine Mayo Clinic Rochester Minnesota USA

**Keywords:** curative, induction, intensive chemotherapy, randomized, venetoclax

Combination therapy with the nucleoside analog cytarabine and anthracycline daunorubicin (“7 + 3”) has shown remarkable durability as the induction regimen of choice for most patients with newly diagnosed non‐promyelocytic acute myeloid leukemia (AML) receiving treatment with curative intent [1]. After four decades, the dominance of “7 + 3” is being challenged by the advent of “lower‐intensity” therapy (LIT) based around the BCL2‐inhibitor venetoclax [2] in patients considered “fit” to withstand intensive chemotherapy [3, 4, 5, 6, 7]. It would be premature to write the obituary of “7 + 3” or its variants, but the emergence of LIT that replaces rather than refines the traditional anthracycline–cytarabine backbone, represents an important inflection point in the history of AML therapies that is worthy of appraisal.

## 1 A Champion Is Crowned

The “7 + 3” combination of cytarabine and daunorubicin emerged from studies in the mid‐20th century, following the recognition that achieving marrow aplasia with induction therapy was required for remission and improved survival in AML [1]. Following early dose‐finding studies, in which treatment was titrated to marrow and peripheral blood cellularity, a 5‐day induction schedule of cytarabine and a second agent, including daunorubicin, was frequently used. However, as many patients required repeated cycles to achieve clearance of marrow blasts, efforts to ensure consistent marrow aplasia through treatment intensification culminated in a regimen incorporating 7 days of cytarabine with 3 days of daunorubicin that achieved complete remission (CR) in 13–15 patients.

These results prompted the Cancer and Leukemia Group B to undertake a larger randomized comparison of 5‐ versus 7‐day cytarabine schedules, as well as two modes of administration—continuous intravenous infusion (100 mg/m^2^/day), versus bolus (100 mg/m^2^ 12 hourly) dosing, alongside 2 or 3 days of daunorubicin (45 mg/m^2^/day) [8]. In this pivotal study published in 1981, the 7‐day induction schedule in de novo AML, including patients with acute promyelocytic leukemia (APL), was associated with higher CR rates and reduced induction mortality compared with the shorter duration of therapy. Although CR rates were higher with infusional cytarabine compared with bolus, this difference was not statistically significant and was potentially influenced by an unexplained decline in responses in the bolus arm later on in the study. An investigation of the mode of cytarabine delivery on survival was confounded by the inclusion of responding patients from all arms of the study in the analyses and the mode of delivery of cytarabine during maintenance therapy. Nevertheless, “7 + 3” offered, for the first time, a consistent path to remission with improved survival in many patients, laying the foundation for therapeutic advances in those “fit” to tolerate intensive treatment.

## 2 Contenders for the Belt—Attempts to Displace “7 + 3”

The ensuing decades saw multiple attempts to improve outcomes through refinement, intensification, or replacement of the “7 + 3” regimen. The repeated failure to identify alternative strategies that meaningfully improved survival was perhaps best captured by the introduction of “SAB” as a purported new standard of care in 2002, a reference to daunorubicin and cytarabine administered two decades later (“same as before”), with improved results attributable not to pharmacological innovation but to advances in supportive care [9]. While tongue‐in‐cheek, the underlying critique of uncontrolled studies and reliance on historical comparators remains both valid and highly relevant during the interpretation of modern single‐arm trials.

Efforts to enhance the “7 + 3” backbone have yielded incremental gains. Dose intensification of daunorubicin and the addition of the antibody–drug conjugate gemtuzumab ozogamicin (GO) have improved outcomes in selected patient groups [1]. Higher daunorubicin doses improved CR rates within single anthracycline‐based induction schedules, but overall survival (OS) gains were dependent on patient age and disease genotype. Similarly, the benefit of GO is context‐dependent, with improved event‐free survival (EFS) observed in broader populations and OS advantages largely restricted to defined biological subgroups.

The relative lack of success in displacing “7 + 3” reflected, in large part, an incomplete understanding of the biological heterogeneity of AML. For many years, AML was approached therapeutically as a single disease entity, with classification based largely on morphology. The advent of systematic cytogenetic classification, followed by molecular genetic discoveries in AML, arising as a by‐product of the Human Genome Project, began to unravel the biological complexity of the disease. By highlighting fundamental genomic differences between AML patients, a framework to explain the variable outcomes in uniformly treated patients was identified, along with the rationale for treatments targeted to genomically defined subgroups.

Unquestionably, progress in AML therapeutics would have been more rapid had biological specimens from earlier clinical trials been routinely banked, enabling retrospective genomic interrogation of well‐annotated cohorts. Recent advances have been confined to specific molecular subsets, with the addition of targeted therapies such as FLT3 inhibitors and GO, integrated with, rather than replacing, the “7 + 3” backbone. Based on biological pathway redundancy within or between leukemic clones—with AML now recognized as a multi‐clonal disease [10] capable of adaptive escape under therapeutic pressure, there remains a strong rationale for continued “broader” targeting of leukemogenic mechanisms alongside consolidation with allogeneic stem cell transplantation (alloSCT) in disease subsets. Accordingly, the liposomal combination of daunorubicin and cytarabine (Vyxeos) used in induction confers an OS advantage over “7 + 3” in patients with clinically or cytogenetically defined secondary AML, restricted to those with myelodysplasia‐related AML who proceed to alloSCT [11]. In addition, intensified induction, incorporating additional nucleoside analogus, improves EFS in unselected patients and confers an OS benefit in *NPM‐1* and *FLT3* mutated disease, compared to daunorubicin and cytarabine combinations [12].

## 3 A New Challenger Emerges

While intensified genotoxic induction therapy of AML can improve disease control in “fit,” patients, the toxicity can be prohibitive in those with comorbidity. For the latter group, the development of venetoclax, particularly in combination with hypomethylating agents such as azacitidine, represents a significant landmark in AML therapeutics. The VIALE‐A study demonstrated an OS advantage for azacitidine plus venetoclax over azacitidine monotherapy in patients considered unsuitable for intensive chemotherapy, with higher response rates and acceptable toxicity [2]. The importance of a venetoclax‐containing therapeutic backbone in AML is further supported by studies in combination with low‐dose cytarabine [13] suggesting that BCL2 inhibition destabilizes a key survival dependency within leukemic cells.

As with “7 + 3” in intensively treated patients, the biological heterogeneity of AML continues to limit the effectiveness of two‐drug, venetoclax‐based LIT across disease subtypes. While responses are particularly favorable in patients with *NPM1* and *IDH* mutations and wild‐type *FLT3* [2, 13], including in those experiencing relapse following intensive chemotherapy or alloSCT [14], patients with monocytic or *FLT3*‐ITD mutated AML have poorer outcomes. Furthermore, data to guide the optimal duration of therapy with venetoclax‐based LIT remain limited, with disease progression or unacceptable toxicity remaining the principal basis for treatment discontinuation.

Nevertheless, the high response rates observed with LIT, by both conventional and measurable residual disease (MRD) assessments, speed of cytoreduction, and feasibility of predominantly outpatient delivery raise two important questions: are the benefits of intensive induction chemotherapy truly unique, and is its toxicity still justified as the price for a cure? Attempts to interrogate this dichotomy are not new. The MRC AML14 trial addressed this question by permitting randomization between intensive (AML14I) and non‐intensive (AML14NI) treatment. However, the vast majority of patients were assigned to one approach by investigators, with only 8 out of 1412 patients undergoing randomization, reflecting constraints within the non‐intensive therapeutic landscape of the time that precluded randomized studies [15]. The effectiveness of venetoclax as a combination partner with hypomethylating agents or “low‐dose” cytarabine now offers a potentially credible alternative to intensive chemotherapy and a realistic opportunity to revisit this question in randomized trials.

## 4 The Bell Has Sounded—Let the Round Begin

Following an anticipated proliferation of retrospective and propensity‐matched analyses comparing outcomes between venetoclax‐based regimens and intensive chemotherapy, a small but growing group of prospective randomized studies has begun to test whether venetoclax‐based LIT can compete with, or even outperform, conventional intensive induction approaches in “fit” patients (Table 1).

**TABLE 1:** Prospective randomized studies comparing venetoclax‐based “lower‐intensity” regimens with intensive chemotherapy as induction strategies in acute myeloid leukemia (AML). The terms “7 + 3” and “3 + 7” are used interchangeably, in accordance with trial nomenclature. For protocol details, dosing and trial design or recruitment, readers should refer to up‐dated publications or trial registries. Reported values have been rounded where appropriate.

Study	Trial ID phase (*n*)	Median age (range)	Eligibility		Induction arms	n	Primary endpoint	ELN 2022 fav/int/adv (%)	CR/CRi (%)	CR (%)	Deaths (%)	Toxicity	alloSCT in CR1	Median follow‐up	1‐year EFS (%)	1‐year OS (%)	Comments
Lu et al. [3]	NCT05177731 Phase IIb (*n* = 188)	43 years (18–59)	≥ 18–59 years (excluding HMA‐treated MDS)	E	VEN‐DEC	94	CRc^a^	48/28/24	89	83	100 days 1	Lower rates of blood product and G‐CSF support, lower rates of grade ≥ 3 pneumonia, febrile neutropenia and sepsis with VEN‐DEC	33%	12.1 months	64	83	Higher CR/CRi in ELN adverse risk and *U2AF*‐mutated disease, lower rates in AML with *RUNX1::RUNX1T1*
				C	IA‐12	94		63/17/20	79	79	100 days 4		39%		63	84	
Fang et al. [4]	NCT06066242 Phase II (*n* = 102)	65 years (60–73)	60–75 years (excluding CBF AML)	E	Aza‐ven	36	EFS	17/33/50	61	47	30 days 3	Similar hematological and non‐hematological toxicity across arms		12.5 months	38	61	EFS/OS benefit with Aza‐ven in ELN adverse risk subgroup
				E	D/IA^b^ + ven	34		35/29/35	62	62	30 days 12		7 (total)		39	57	
				C	D/IA	32		25/25/44	41	38	30 days 16				29	44	
Fathi et al. [5]	NCT04801797 Phase II (*n* = 172)	64 years (23–79)	≥ 18 years (excluding CBF, *FLT3*‐mut, *NPM1*‐mut < 60y)	E	Aza‐ven	86	EFS	15/17/67	70	56	30 days/60 days Nil/Nil	Lower rates of Grade ≥ 3 infection and bleeding with Aza‐ven	60%	21.9 months	53	22^c^	PROMS and healthcare utilization benefits with Aza‐ven
				C	“3 + 7” or CPX‐351	86		11/13/77	52	49	30 days/60 days 4/5		40%		36	19^c^	
VINCENT [6]	NCT05904106 Phase II		18–70 years with *NPM1*‐mut, *FLT3*‐wt AML	E	Aza‐ven	Target 146	Modified EFS^a^, ^d^	Recruiting									
				C	“7 + 3” + GO												
VICTOR [7]	ISRCTN15567173 Phase II		≥ 55 years with *NPM1*‐mut, *FLT3*‐wt AML	E	ven + LDAC	Target 156	Molecular EFS^a^, ^e^	Recruiting									
				C	DA + GO												
QUIZZICAL	UK AML RN study Phase II		≥ 50 years with *FLT3*‐ITD‐mutated AML	E	Quizartinib + Aza‐ven	Target 176	OS	In set‐up									
				C	“7 + 3” + Quizartinib												

Abbreviations: alloSCT, allogeneic stem cell transplantation; Aza‐ven, azacitidine plus venetoclax; C, control arm; CBF, core‐binding factor; CR, complete remission; CRc, composite complete remission (defined as CR + CRi); CRi, complete remission with incomplete count recovery; D/IA, daunorubicin or idarubicin (3 days) with cytarabine (7 days); DA, daunorubicin and cytarabine in a “3 + 10” schedule; E, experimental arm; EFS, event‐free survival; ELN, European LeukemiaNet [risk groups presented as favorable (fav)/intermediate (int)/adverse (adv)]; G‐CSF, granulocyte colony‐stimulating factor; GO, gemtuzumab ozogamicin; HMA, hypomethylating agent; IA‐12, idarubicin and cytarabine; IC, intensive chemotherapy; LDAC, low‐dose cytarabine; NR, not reached; OS, overall survival; PROMS, patient‐reported outcome measures; VEN‐DEC, venetoclax plus decitabine.

^a^ Non‐inferiority.

^b^ “2 + 5” schedule of daunorubicin or idarubicin for 2 days and cytarabine for 5 days.

^c^ Median OS.

^d^ Primary induction failure, hematologic relapse, molecular failure, or death.

^e^ Failure to achieve CR/CRi after two cycles, molecular persistence, progression or relapse requiring treatment change, morphological relapse or death.

Early signals from these studies are provocative, albeit preliminary. In a Phase 2b study from Suzhou (NCT05177731) [3], venetoclax and decitabine (VEN‐DEC) were non‐inferior to idarubicin and cytarabine in younger adults with de novo AML, with higher composite remission rates (89% vs. 79%) and similar rates of MRD negativity. Notably, responses appeared particularly encouraging in adverse‐risk genetic disease, including in selected molecular subsets, although outcomes varied across genotypes, with inferior responses observed in core‐binding factor (CBF) AML. Toxicity profiles favored VEN‐DEC, with lower rates of severe infection and febrile neutropenia, albeit with comparable hematological recovery.

A second randomized Phase 2 study (NCT06066242) [4] in older adults (60–75 years) comparing venetoclax–azacitidine, intensive chemotherapy, and a hybrid approach incorporating venetoclax demonstrated no significant differences in EFS or OS across arms at early follow‐up. However, in patients with adverse‐risk genetics, outcomes appeared more favorable with venetoclax–azacitidine, with comparable toxicity. Rates of transplantation were low across all arms of the study, and as with the study from Suzhou, all patients in remission received intensive consolidation.

Against this backdrop, the Phase 2 PARADIGM study (NCT04801797) [5] represents the most direct comparison of LIT with conventional intensive induction in “fit” adults with AML, the majority of whom had adverse‐risk disease by ELN 2022. In this study, venetoclax–azacitidine improved EFS and achieved higher response rates than intensive chemotherapy with “7 + 3” or Vyxeos, with lower early mortality, improved quality of life, and reduced healthcare utilization. A greater proportion of patients receiving venetoclax‐based therapy proceeded to alloSCT. These early observations suggest that a venetoclax‐based LIT approach may not only match, but potentially surpass, intensive chemotherapy in a population traditionally considered suitable for intensive induction. However, with a high representation of patients predicted to fare poorly with intensive chemotherapy, the enrolled cohort may represent a selected population in whom equipoise between intensive and non‐intensive approaches was already uncertain.

Ultimately, selection of induction intensity and strategy should be guided by biologically defined AML subgroups, and embedded within randomized evaluations of intensive chemotherapy versus LIT‐based approaches to define future treatment pathways. This approach is being explored in ongoing and planned studies. VINCENT [6] and VICTOR [7], for example, are assessing venetoclax‐based strategies with azacitidine or low‐dose cytarabine, respectively, against intensive chemotherapy in *NPM1*‐mutated, *FLT3*‐wild‐type (*NPM1* ^mut^/*FLT3* ^WT^) AML, a subgroup enriched for venetoclax sensitivity. The QUIZZICAL study (Professor Charles Craddock, personal communication) seeks to extend this paradigm to *FLT3*‐ITD mutated disease for “fit” patients by incorporating targeted inhibition using a triplet of venetoclax, azacitidine and the FLT3 inhibitor quizartinib as the investigational arm. Collectively, these trials signal a shift toward biology‐informed evaluation of induction strategies, moving beyond a binary distinction of “fitness” in unselected patients.

## 5 A New Champion Across Categories, or Within Each Category?

Detailed analyses and longer follow‐up from recently completed studies will be essential to inform larger trials and define the optimal induction strategy for specific biological subtypes of AML. At present, in “fit,” non‐trial patients with “favorable risk” AML, such as those characterized by CBF re‐arrangements, *NPM1* ^mut^/*FLT3* ^WT^, or in‐frame mutations affecting the basic leucine zipper region of *CEBPA*, the “standard‐of‐care” should remain intensive induction with a “7 + 3” (or intensified) backbone. This treatment should be coupled with an MRD‐directed approach to identify patients who will benefit from alloSCT in CR1 and those desiring a finite duration of therapy. VINCENT [6] and VICTOR [7] could reshape the treatment pathway in *NPM1* ^mut^/*FLT3* ^WT^ AML, particularly if an MRD‐directed time point can reliably predict the safety of treatment discontinuation—potentially enabling entire replacement of the “7 + 3” backbone, analogous to the use of arsenic trioxide and all‐trans retinoic acid in APL. Emerging determinants of outcome, including baseline disease features and MRD kinetics across both arms of these trials, could further optimize treatment intensity and type, with poorer‐risk disease stratified for additional targeting with menin inhibitors [16].

In contrast to this biologically stratified pharmacological approach, in “adverse‐risk” AML, where alloSCT remains the only realistic prospect of cure, induction therapy is largely “preparative” for transplant‐eligible patients. Regimen selection should therefore prioritize rapid disease control with minimal toxicity while preserving fitness for timely alloSCT. Accordingly, many clinicians may view recent randomized data on induction intensity [3, 4, 5] as practice‐affirming. However, as “adverse risk” represents a prognostic rather than a biological categorization, a uniform treatment backbone is unlikely to be appropriate for all patients within this subgroup. In addition, given the use of age‐ and treatment‐intensity‐agnostic risk stratification [17, 18, 19] across recent intensive‐versus‐LIT trials [3, 4, 5], it is important to consider whether the intensive chemotherapy arm underperformed or LIT overachieved relative to contemporary studies [2, 20, 21]. Notwithstanding these results, efforts to identify novel management strategies for “ultrahigh risk” AML subsets, for example, with *TP53* mutations or *MECOM* re‐arrangements, remain essential, as sustaining the durability of the modest disease responses achieved with current therapies, whether intensive or LIT‐based, remains extremely challenging.

A further challenge in identifying the optimal induction intensity and type lies in intermediate‐risk AML, where a proportion of patients is cured with “7 + 3” or its variants, without the need for alloSCT. In this group, a baseline relapse risk of ≥ 50%, independent of post‐induction MRD, may be reduced by alloSCT in patients with an HCT‐CI ≤ 2, albeit at the cost of non‐relapse mortality approaching 20%–30% [22]. Whether the incorporation of targeted agents in molecularly defined subsets of intermediate‐risk disease, within “7 + 3,” intensified, or venetoclax‐based backbones, could enable more selective, MRD‐directed use of transplantation as consolidation, remains an open question. Signals from studies incorporating agents such as FLT3 or IDH1 inhibitors alongside intensive chemotherapy or venetoclax‐based LIT backbones, or from randomized comparisons between intensive and LIT, as proposed in QUIZZICAL, would be particularly informative for defining the optimal management strategy. Pending maturation of ongoing studies incorporating menin inhibitors in *KMT2A*‐rearranged AML [16], randomized comparisons between intensive and LIT backbones containing these agents, coupled with MRD‐directed stratification for alloSCT, represent an exciting prospect for this disease subtype and for intermediate‐risk AML subsets characterized by *HOXA* and *MEIS1* upregulation that may be susceptible to menin inhibition.

The excitement of these drug combinations; however, needs to be tempered with the potential for toxicity: as with the developmental days of “7 + 3,” the toxicity profile of newer multi‐agent therapy is only beginning to be understood [23]. Crucially, any gains in efficacy with LIT must not come at the cost of toxicity comparable to intensive induction, particularly as preservation of fitness remains central to successful transplantation. In this context, an important point for consideration would be whether the transplant strategy needs to be adapted to the intensity of induction therapy that a patient has received.

## 6 Delivering the Knock‐Out Blow—Optimizing alloSCT Outcomes

Given the central importance of alloSCT as the only strategy that improves survival for many patients, efforts have focused on identifying sensitive determinants of outcome and interventions that optimize the balance between relapse risk and non‐relapse mortality across a broader patient population. In this context, MRD assessment is central to defining the depth of disease control, as patients with detectable MRD prior to alloSCT are generally recognized to have a higher risk of relapse and inferior survival [24, 25]. However, this relationship is not absolute. A significant proportion of MRD‐positive patients do not experience relapse post‐alloSCT [25, 26], particularly those who achieve MRD negativity following transplantation, either through the intensity of conditioning [24], possibly in a genotype‐dependent manner [27], or through conversion to a fully donor‐derived T‐cell compartment [28]. The post‐alloSCT MRD status may therefore be a stronger determinant of relapse risk than pretransplant MRD status.

These observations, derived largely from patients undergoing alloSCT after intensive induction, suggest that pretransplant MRD positivity is a dynamic variable that can be therapeutically modified by the conditioning regimen, donor‐derived immune reconstitution, posttransplant maintenance, or other immune‐based strategies. Two recent randomized trials have suggested the equivalence of MRD‐negative states following intensive chemotherapy and LIT [3, 4], helping to allay concerns regarding the depth of remission achieved prior to alloSCT; the ability to further modulate disease burden after transplantation should provide additional reassurance.

Nevertheless, important caveats remain. Experience in B‐cell acute lymphoblastic leukemia suggests that even among MRD‐negative patients, additional survival benefit is derived from immunotherapeutic strategies [29], implying that MRD assessments, particularly those that are flow cytometry‐based, may not fully capture the depth of residual disease. Longer follow‐up from pivotal studies such as PARADIGM [5], with particular focus on the cumulative incidence of posttransplant relapse in relation to pretransplant MRD negativity (which has typically shown less dependence on conditioning intensity in patients receiving intensive induction) [24], adjusted for disease genotype, will be essential to establish whether true equivalence exists between MRD‐negative states achieved following intensive and LIT. At the other end of the spectrum, a subset of patients with refractory or relapsed AML following intensive induction can achieve durable survival following transplantation despite incomplete cytoreduction at the time of graft infusion [30]. Whether this observation can be extended to patients achieving a similar disease response following LIT, without the need for “triplet” combinations, would be of considerable interest.

Reconciling these seemingly opposing observations requires a broader perspective. The level of disease control necessary for successful transplantation is unlikely to be uniform; rather, it is likely to reflect an interplay between disease biology, conditioning intensity, and the kinetics of immune reconstitution. Some patients may benefit from deeper cytoreduction during conditioning, others from rapid establishment of donor‐derived immunity, and others still from posttransplant maintenance strategies that sustain disease control, at least until the development of an effective graft‐versus‐leukemia effect. In this context, induction intensity may indeed shape posttransplant outcomes through the “state” in which it delivers the patient to transplantation: the burden and clonal architecture of residual leukemia, together with the preservation or erosion of physiological reserve. A systematic characterization of these parameters will help define the optimal alloSCT strategy required for durable disease control.

## 7 Conclusion

**FIGURE 1:**
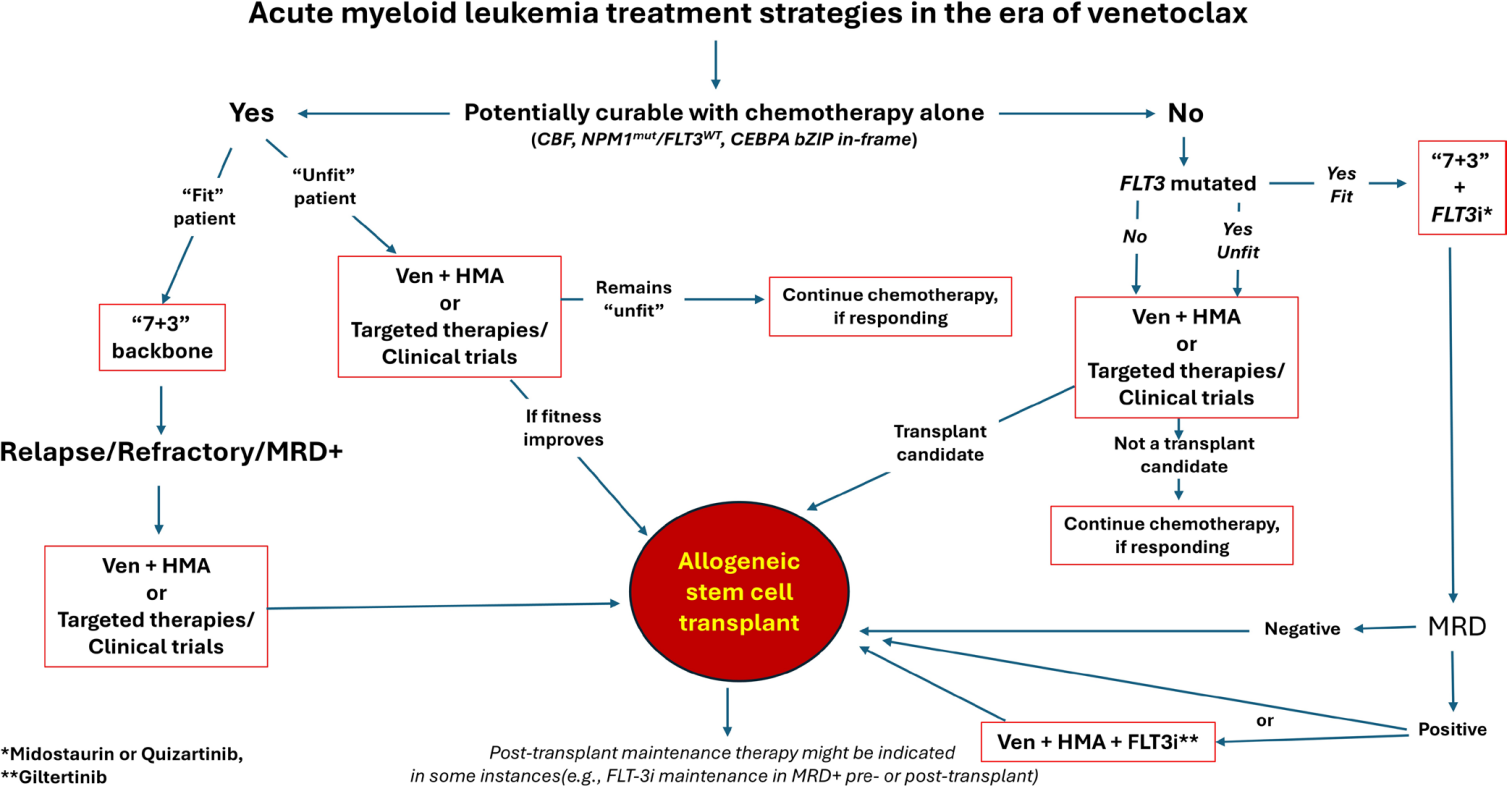
Treatment algorithm for newly diagnosed acute myeloid leukemia (AML) in the era of venetoclax (Ven)‐based lower‐intensity therapies. “7 + 3” remains the reference induction backbone for disease subsets potentially curable with intensive chemotherapy but may increasingly be modified according to disease biology and measurable residual disease (MRD) status. Incorporation of targeted therapies such as FLT3 inhibitors (FLT3i) within the treatment pathway, or replacement of the intensive chemotherapy backbone with venetoclax–hypomethylating agent (HMA)‐based therapy, represents an emerging area of interest that places both disease biology and patient fitness at the center of the treatment paradigm. [Color figure can be viewed at wileyonlinelibrary.com]

The emerging evidence suggests that the future of AML therapy is unlikely to be defined by a single therapeutic backbone (Figure 1). Instead, it will depend on a more nuanced alignment of induction intensity and type, with disease biology and patient‐specific factors, integrated with alloSCT in selected patients. Indeed, the prospect of identifying an undisputed “therapeutic champion” for each biologically defined disease subset, rather than across the categories, offers a more rational and optimistic framework for patients and physicians alike.

## Author Contributions

S.T. and A.T. authors contributed equally to the conception, development, writing, and revision of the manuscript.

## Funding

The authors have nothing to report.

## Ethics Statement

The authors have nothing to report.

## Consent

The authors have nothing to report.

## Conflicts of Interest

The authors declare no conflicts of interest.

## Data Availability

The authors have nothing to report.
